# Thyroid storm associated with Graves' disease covered by diabetic ketoacidosis: A case report

**DOI:** 10.1186/1756-6614-4-8

**Published:** 2011-04-14

**Authors:** Erika Osada, Naoki Hiroi, Mariko Sue, Natsumi Masai, Ryo Iga, Rika Shigemitsu, Reiko Oka, Masahiko Miyagi, Kaoru Iso, Koji Kuboki, Gen Yoshino

**Affiliations:** 1 Division of Diabetes, Metabolism and Endocrinology, Department of Internal Medicine (Omori), Toho University School of Medicine, Tokyo, Japan

## Abstract

**Background:**

Thyroid storm is a condition in which multiple organ dysfunction results from failure of the compensatory mechanisms of the body owing to excessive thyroid hormone activity induced by some factors in patients with thyrotoxicosis. While diabetic ketoacidosis (DKA) is an important trigger for thyroid storm, simultaneous development of DKA and thyroid storm is rare.

**Case presentation:**

A 59-year-old woman with no history of either diabetes mellitus or thyroid disease presented to our hospital because of developing nausea, vomiting and diarrhea for 2 days. Physical examination showed mild disturbance of consciousness, fever, and tachycardia. There were no other signs of thyrotoxicosis. Laboratory studies revealed elevation of random blood glucose and glycosylated hemoglobin, strongly positive of urine acetone, and metabolic acidosis. Since DKA was diagnosed, we initiated the patient on treatment with administration of insulin and adequate fluid replacement. Although the hyperglycemia and acidosis were immediately relieved, the disturbance of consciousness and tachycardia remained persistent. Levels of FT3 and FT4 were extremely high and TSH was below the detectable limit. TRAb was positive. The thyroid storm score of Burch & Wartofsky was 75/140, and the thyroid storm diagnostic criteria of the Japan Thyroid Association were satisfied. Oral administration of thiamazole, potassium iodide and propranolol resulted in immediate relief of the tachycardia.

**Discussion:**

We encountered a case of thyroid storm associated with Graves' disease covered by DKA. Thyroid storm and DKA are both potentially fatal, and the prognosis varies depending on whether or not these conditions are detected and treated sufficiently early. The thyroid storm diagnostic criteria prepared in 2008 by the Japan Thyroid Association are very simple as compared to the Burch & Wartofsky scoring system for thyroid storm. The Japanese criteria may be useful in the diagnosis of this condition since they enable clinicians to identify a broad range of cases with thyroid storm. When dealing with cases of DKA or thyroid storm, it seems essential to bear in mind the possibility of the coexistence of these two diseases.

## Introduction

Thyroid storm is a condition involving failure of multiple organs arising from collapse of the compensatory mechanism for excessive thyroid hormone activity triggered by factors associated with thyrotoxicosis. The incidence of thyroid storm is reported to be less than 10% in patients hospitalized for thyrotoxicosis, but the fatality rate is reportedly 20 - 30%, if this disease is not treated promptly [1,2]. Diabetic ketoacidosis (DKA) is a severe metabolic disorder arising from deficient insulin activity. It is reported that DKA develops in 5 out of 1000 diabetic patients annually from Taiwan [3]. If this disease follows a severe course due to lack of appropriate diagnosis and treatment, the patient may die in the comatose state, with the death rate reported to be 2% or less [4]. We recently encountered a case in which thyroid storm and DKA developed simultaneously.

### Case report

The patient was a 59-year-old woman with chief complaints of nausea and vomiting. Four months prior to presentation, malaise and anorexia manifested but were left untreated. She had been free of signs of diabetes, eg, thirst, polydipsia, and polyuria, and signs of Graves' disease, eg, hyperhidrosis, tremor, neck swelling, and exophthalmos. Two days before presentation, she developed nausea, vomiting and diarrhea. At that time, acute gastroenteritis was suspected, and oral medication was prescribed. However, frequent vomiting persisted thereafter. At that time, a high blood glucose level and positive urinary acetone were noted. She was thus suspected of having DKA and was brought by ambulance to our hospital. When first examined at our hospital, the patient was 159 cm in height, weighed 64.0 kg, and had a body mass index of 25.3 kg/m^2^, blood pressure 140/62 mm Hg, heart rate 170/min (regular), and temperature 38.2°C. Consciousness level was E3, V3, M6 and total 12 points according to the Glasgow Coma Scale. The skin showed low turgor and was not moist. No tremor was observed. Upper eyelid edema was present, but there was no marked exophthalmos. Slight erosive swelling of the thyroid was noted. There were no chest abnormalities. Borborygmus was enhanced but no other abnormalities were noted in the abdomen. There were no neurological abnormalities.

Biochemically, blood glucose and glycosylated hemoglobin were markedly elevated (336 mg/dL, 10.9%), while total cholesterol (126 mg/dL) and neutral fat (72 mg/dL) were at the lower limits of their normal ranges. Mild hepatic dysfunction, hyperuricemia, and mild hypoproteinemia were noted. Urine acetone was strongly positive. On blood gas analysis, acidosis (pH 7.257) was noted, accompanied by an expanded anion gap, and blood ketone body elevation (predominantly β-hydroxybutyric acid) was also seen (β-hydroxybutyric acid 5920 mmol/L, acetoacetic acid 2101 mmol/L). Anti-glutamic acid decarboxylase antibody and anti-insulinoma-associated protein-2 antibody were negative, and normal reaction of insulin was observed to glucagon loading. Chest X-ray disclosed only mild cardiomegaly, and electrocardiography revealed sinus tachycardia. After heart rate improvement, echocardiography disclosed no evident abnormalities.

### Clinical course

Based on the diagnosis of DKA, the patient was fasted and received a massive physiological saline infusion for correction of dehydration, accompanied by initiation of insulin therapy. Her blood glucose level dropped rapidly, but the slightly reduced consciousness level (the Glasgow Coma Scale: E4, V4, M6 and total 14 point), tachycardia (150/min), and fever (38.0°C) persisted. Because tachycardia could not be explained by DKA-associated dehydration alone, thyroid disease was added to the differential diagnosis. Free triiodothyronine (FT3) and free thyroxin (FT4) were 26.28 pg/mL and >7.77 ng/dL, respectively, and thyroid stimulating hormone (TSH) was below the detectable limit. TSH receptor antibody (TRAb) was positive (24.9 IU/L). Thyroid ultrasonography revealed diffuse swelling of the thyroid gland, a reduced internal echo level, and increased internal blood flow. Although the patient had DKA as a complication, the thyroid storm score of Burch & Wartofsky [5] was 75/140, and the thyroid storm diagnostic criteria of the Japan Thyroid Association (JTA) [6] were satisfied. Thyroid storm associated with Graves' disease was thus considered highly probable in this case, and treatment with 15 mg/day of thiamazole, 100 mg/day of potassium iodide and 30 mg/day of propranolol was started. The following day, the fever subsided to approximately 37°C and tachycardia also showed alleviation.

Two months later, thyroid function had been normalized. The patient is still being managed with oral thiamazole therapy. Regarding diabetes, this case initially required a maximum insulin aspart dose of 52 units/day. Blood glucose control was achieved with 14 units of insulin aspart plus 16 units of insulin glargine. The patient was suspected of having type 2 diabetes mellitus because all islet-related antibodies were negative and delta C-peptide (CPR) level after glucagon loading (6 min after glucagon loading - before glucagon loading) was 1.1 ng/mL. However, the honeymoon phenomenon in the type 1 diabetes mellitus could not be ruled out. One year later, the dosage of insulin was decreased gradually to 12 units in total, and the improvement in ability of insulin secretion was assessed by glucagon loading (delta CPR: 1.6 ng/mL). Therefore, the patient was finally diagnosed as having type 2 diabetes mellitus although insulin administration was required.

## Discussion

Our present case had thyroid storm associated with Graves' disease covered by DKA. She lacked signs of thyrotoxicosis and was strongly suspected of having DKA on the basis of clinical findings and test data. Upon admission to the hospital, she was diagnosed as having DKA and was treated accordingly. Although it has been reported that detection of thyroid storm tends to be delayed in patients with DKA because of suppression of fever [4,7,8], relatively low thyroid hormone levels [9], in the present case, none of these findings was obtained. In this case, the diagnosis of thyroid storm was probably delayed because the symptoms of DKA resembled those of thyroid storm and because no symptoms of Graves' disease were observed before the onset of thyroid storm. However, the presence of thyrotoxicosis was suspected in a relatively early stage in this case based on the following findings: (1) symptoms of central nervous system involvement, as well as tachycardia and fever, not alleviated despite correction of the dehydration associated with DKA; and (2) a relatively low total cholesterol level. Thus, treatment with an iodine preparation, β-blocker, and thiamazole was initiated relatively quickly, and this early treatment probably accounts for the favorable outcome of our patient. Generally, large amounts of thiamazole, potassium iodide, and prednisolone are required for the treatment of thyroid crisis; however, thyroid function was restored with a few doses of these medications in our case. This could be one of the reasons why DKA could deteriorate the thyroid storm symptoms.

An interesting point in this case is whether or not the patient really had thyrotoxicosis at the time of admission. When initially examined, she showed laboratory data of thyrotoxicosis and was given a score of 75 according to the thyroid storm score of Burch & Wartofsky [5]. Thus, the patient seemed to satisfy the thyroid storm diagnostic criteria. Furthermore, this patient also apparently satisfied the thyroid storm diagnostic criteria prepared by the JTA (Table 1) [6], because she had signs of thyrotoxicosis, accompanied by a reduced consciousness level (a central nervous system symptom), tachycardia, fever, and gastrointestinal symptoms. Even after correction of dehydration and blood glucose level, the Burch & Wartofsky score was 45 and symptoms of central nervous system involvement, fever, and tachycardia persisted, thus satisfying the Japanese diagnostic criteria. Treatment with potassium iodide rapidly alleviated clinical symptoms, suggesting that thyroid storm had already been present on admission. The thyroid storm diagnostic criteria prepared in 2008 by the JTA are very simple as compared to the thyroid storm score of Burch & Wartofsky. Thyroid storm has a high fatality rate if it is not appropriately diagnosed and treated. The Japanese criteria may be useful in the diagnosis of this condition since they enable clinicians to identify a broad range of cases with thyroid storm.

**Table 1:** Diagnostic criteria for thyroid storm of Japan Thyroid Association and Japan Endocrine Society

Essential criterion	Presence of thyrotoxicosis (elevation of free T3 and/or T4)	
Symptoms	1.	Symptoms involving the central nervous system
	2.	Fever (≥ 38°C)
	3.	Tachycardia (≥ 130/min)
	4.	Symptoms of heart failure
	5.	Gastrointestinal symptoms
Cases definitely diagnosed as having thyroid storm		
Satisfaction of the essential criterion and at least one of the following criteria		
	a.	Central nervous system symptoms + one or more of the other systems, or
	b.	3 or more symptoms other than those of central nervous system.
Cases suspected of having thyroid storm		
	a.	Satisfaction of essential criterion + 2 symptoms other than those of the central nervous system, or
	b.	Satisfaction of essential criterion is not confirmed, but positive history of thyroid disease + exophthalmos + goiter are present and criterion a or b for definite cases is satisfied.

The malaise and anorexia, which this patient had been experiencing for 4 months, were not inconsistent with the symptoms of diabetes mellitus. However, whether or not Graves' disease was present 4 months prior to presentation is unknown. Which developed earlier, the diabetes or Graves' disease, also remains unknown. Regarding the influence of Graves' disease on diabetes mellitus, there is a general agreement that the deterioration of glucose intolerance, including diabetes mellitus, is observed under a hyperthyroid state [10]. Graves' disease has been reported to increase the intestinal absorption of carbohydrates [11] and production of hepatic glucose from glycogen due to hepatic insulin resistance [12,13]. Hyperthyroid patients show marked insulin resistance to oral glucose tolerance test that resolves with the treatment of hyperthyroidism [14]. Glucose uptake in the skeletal muscle is known to be lower in hyperthyroid patients than in euthyroid patients; this difference is also attributable to insulin resistance [15,16]. The ability of insulin to suppress hormone-sensitive lipase --an enzyme responsible for adipocyte fatty acid release, which facilitates β-oxidation and ketone body production in the liver-- is also known to be affected by hyperthyroidism-induced insulin resistance [15]. The increase in the glomerular filtration rate in hyperthyroidism leads to an increased insulin clearance and hence a decreased insulin level [17]. In the hyperthyroid state, the ratio of C-peptide to proinsulin is low [18], suggesting an increase in the rate of biologically inactive insulin precursor release. These findings suggest that thyroid hormones are associated with the onset and aggravation of diabetes mellitus. In addition, β-cell function is known to be lower in Japanese individuals than in other ethnic groups [19], and age-related β-cell dysfunction and/or reduction of insulin sensitivity were usually observed [20,21]. It seems probable that these factors aggravated the underlying abnormal glucose metabolism (ie, diabetes mellitus) and intensified insulin deficiency, serving as a factor responsible for the onset of DKA in this case. Meanwhile, thyroid storm is usually precipitated by infection, trauma and surgical emergencies, or operations and less commonly by radiation thyroiditis, DKA, toxemia of pregnancy, or parturition [22,23]. Therefore DKA cannot be ignored as a factor triggering the onset of thyroid storm [24]. It seems likely that DKA aggravated her underlying Graves' disease, triggering the onset of thyroid storm.

As illustrated above, we recently encountered a DKA case with Graves' disease complicated by thyroid storm. DKA and thyroid storm are both potentially fatal, and the prognosis varies depending on whether or not these conditions are detected and treated with sufficient earliness. When dealing with cases of DKA or thyroid storm, it seems essential to bear in mind the possibility of the coexistence of these two diseases. The present report is presented from this perspective.

### Consent

Written informed consent was obtained from the patient's relatives for publication of this case report. A copy of the written consent is available for review by the Editor-in-Chief of this journal.

## Competing interests

The authors declare that they have no competing interests.

## Authors' contributions

EO wrote the first draft. NM, RI, RS and RO collected information on the patient. MS, MM and KI did the literature searches. NH wrote the final manuscript and made appropriate revisions. NH, KK and GY were in a position of leadership for the patient. All authors read through and approved the final manuscript.
